# OCT1-dependent uptake of structurally diverse pyrrolizidine alkaloids in human liver cells is crucial for their genotoxic and cytotoxic effects

**DOI:** 10.1007/s00204-023-03591-4

**Published:** 2023-09-07

**Authors:** Manuel Haas, Gabriel Ackermann, Jan-Heiner Küpper, Hansruedi Glatt, Dieter Schrenk, Jörg Fahrer

**Affiliations:** 1 Division of Food Chemistry and Toxicology, Department of Chemistry, RPTU Kaiserslautern-Landau, Erwin-Schroedinger-Str. 52, 67663 Kaiserslautern, Germany; 2 Division of Molecular Cell Biology, Department of Environment and Nature Science, Brandenburg University of Technology Cottbus-Senftenberg, 01968 Senftenberg, Germany; 3 Department Food Safety, German Federal Institute for Risk Assessment (BfR), Max-Dohrn-Strasse 8-10, 10589 Berlin, Germany; 4 Department of Nutritional Toxicology, German Institute of Human Nutrition (DIfE), Potsdam-Rehbrücke, Arthur-Scheunert-Allee 114-116, 14558 Nuthetal, Germany

**Keywords:** Pyrrolizidine alkaloids, Cytotoxicity, Genotoxicity, OCT1, Transport, Primary human hepatocytes, γH2AX, p53

## Abstract

Pyrrolizidine alkaloids (PAs) are important plant hepatotoxins, which occur as contaminants in plant-based foods, feeds and phytomedicines. Numerous studies demonstrated that the genotoxicity and cytotoxicity of PAs depend on their chemical structure, allowing for potency ranking and grouping. Organic cation transporter-1 (OCT1) was previously shown to be involved in the cellular uptake of the cyclic PA diesters monocrotaline, retrorsine and senescionine. However, little is known about the structure-dependent transport of PAs. Therefore, we investigated the impact of OCT1 on the uptake and toxicity of three structurally diverse PAs (heliotrine, lasiocarpine and riddelliine) differing in their degree and type of esterification in metabolically competent human liver cell models and hamster fibroblasts. Human HepG2-CYP3A4 liver cells were exposed to the respective PA in the presence or absence of the OCT1-inhibitors d-THP and quinidine, revealing a strongly attenuated cytotoxicity upon OCT1 inhibition. The same experiments were repeated in V79-CYP3A4 hamster fibroblasts, confirming that OCT1 inhibition prevents the cytotoxic effects of all tested PAs. Interestingly, OCT1 protein levels were much lower in V79-CYP3A4 than in HepG2-CYP3A4 cells, which correlated with their lower susceptibility to PA-induced cytotoxicity. The cytoprotective effect of OCT1 inhibiton was also demonstrated in primary human hepatocytes following PA exposure. Our experiments further showed that the genotoxic effects triggered by the three PAs are blocked by OCT1 inhibition as evidenced by strongly reduced γH2AX and p53 levels. Consistently, inhibition of OCT1-mediated uptake suppressed the activation of the DNA damage response (DDR) as revealed by decreased phosphorylation of checkpoint kinases upon PA treatment. In conclusion, we demonstrated that PAs, independent of their degree of esterification, are substrates for OCT1-mediated uptake into human liver cells. We further provided evidence that OCT1 inhibition prevents PA-triggered genotoxicity, DDR activation and subsequent cytotoxicity. These findings highlight the crucial role of OCT1 together with CYP3A4-dependent metabolic activation for PA toxicity.

**Supplementary Information:**

The online version contains supplementary material available at 10.1007/s00204-023-03591-4.

## Introduction

Pyrrolizidine alkaloids (PAs) are phytotoxins with a high structural diversity formed in 3% of all flowering plant species worldwide (Chen et al. 2010). PAs are frequently found as contaminants in plant-based food like herbal teas or spices (Chen et al. 2010; Fu et al. 2004). Several cases of acute and subacute PA intoxications were reported previously in humans or animals due to consumption of contaminated food or PA producing plants, which were characterized by hepatomegaly, ascites, hepatic sinusoidal obstruction syndrome (HSOS) and acute liver failure (Moreira et al. 2018; Teschke et al. 2021). More recently, exposure to PAs has also been linked to human liver cancer formation in Asian countries (He et al. 2021a).

Chemically, PAs are composed of the necine base 1-hydroxymethylpyrrolizidine, which can be esterified with one or two necine acids, resulting in the formation of PA monoesters, open-chained diesters or cyclic diesters (He et al. 2021b; Schrenk et al. 2020). PAs with a 1,2-unsaturated necine base are known to be hepatotoxic, genotoxic and possibly carcinogenic, which is attributable to their bioactivation by cytochrome P450 (CYP) monooxygenases (mostly CYP3A4, but also CYP2B subfamilies) in the liver (Chen et al. 2010; Edgar et al. 2015; Prakash et al. 1999). This biotransformation step gives rise to dehydro-pyrrolizidine derivatives (dehydro-PAs) and, upon hydrolysis, to (±)-6,7-dihydro-7-hydroxy-1-hydroxymethyl-5H-pyrrolizine (DHP), which both react with DNA and proteins (Edgar 2014; Fu 2017). A plethora of studies performed in different liver cell models including human primary hepatocytes demonstrated a structure–toxicity relationship for PAs, supporting the concept of grouping PAs into potency classes (Allemang et al. 2018; Gao et al. 2020; Haas et al. 2023; Hadi et al. 2021; Lester et al. 2019; Louisse et al. 2019; Merz and Schrenk 2016; Rutz et al. 2020).

An important process for their hepatotoxic mode of action is the uptake of PAs into hepatocytes as well as the efflux of PAs or their metabolites. Hepatocytes express an array of influx and efflux transporters belonging to the solute carrier (SLC) and ATP-binding cassette (ABC) superfamily, respectively (Nigam 2015). The SLC22A family comprise the organic cation transporters (OCTs), which mediate the uptake of cationic compounds such as nutrients, endogenous substrates and active pharmaceutical ingredients into cells (Koepsell 2021). The main members are OCT1 (SLC22A1), OCT2 (SLC22A2) and OCT3 (SLC22A3), which share common substrates and can therefore substitute for each other (Brosseau and Ramotar 2019). However, these influx transporters differ in the tissue-specific expression levels. OCT1 is mainly expressed in hepatocytes, whereas OCT2 is primarily found in renal tubular cells. OCT3 expression is detected in many tissues (Brosseau and Ramotar 2019; Koepsell 2021). The OCT substrates include the prototypical chemical compound 1-methyl-4-phenyl-pyridinium (Koepsell 2013), endogenous compounds such as acetylcholine and catecholamine (Breidert et al. 1998; Nakata et al. 2013) as well as pharmaceuticals including metformin and imatinib (Chen et al. 2014; White et al. 2006). First evidence for the involvement of OCT1 in the uptake of PAs was obtained in a study with the cyclic PA diester monocrotaline, which was shown to be taken up into genetically engineered MDCK cells with human OCT1 expression and into primary rat hepatocytes (Tu et al. 2013). This was subsequently confirmed for retrorsine, which also belongs to the group of cyclic PA diesters (Tu et al. 2014). Two more recent studies performed in human HepaRG hepatoma cells revealed that both OCT1 and Na^+^/taurocholate co-transporting polypeptide (SLC10A1) are involved in the hepatocellular uptake of retrorsine and senescionine, which are both cyclic PA diesters (Enge et al. 2021, 2022).

Up to now little information is available on the impact of the chemical structure (PA monoester vs. open-chained diester vs. cyclic diester) on the OCT1-mediated uptake into hepatocytes. Therefore, we selected three structurally diverse PAs (heliotrine, lasiocarpine and riddelliine) and analyzed their toxicity in human HepG2-CYP3A4 liver cells, primary human hepatocytes (PHH) and V79-CYP3A4 Chinese hamster fibroblasts. The impact of OCT1 was studied using the pharmacological inhibitors d-tetrahydropalmatine (d-THP) and quinidine. First, the cytotoxicity of the three PAs was investigated using the resazurin reduction assay in the absence or presence of OCT1 inhibitors. Subsequently, the genotoxic effects of the three selected PAs were determined with and without OCT1 inhibition using western blot analysis of the DNA damage markers γH2AX and p53. Furthermore, the DNA damage response markers pCHK1 and pCHK2 were assessed upon PA exposure in the absence or presence of OCT1 inhibitors.

## Results

### PA triggered cytotoxicity is rescued by pharmacological OCT1 inhibitors in human liver cells

To study the role of OCT1 in the hepatocellular uptake of the selected PAs (SI Fig. 1), we used quinidine and d-THP as established OCT1 inhibitors (Ingoglia et al. 2015; Tu et al. 2013). First, the cytotoxicity of these pharmacological OCT inhibitors was assessed in wild-type HepG2 cells and genetically engineered HepG2 cells with CYP3A4 overexpression (SI Fig. 2). To this end, the cells were exposed to increasing OCT inhibitor concentrations (0–1000 µM) for 24 h and viability was determined by the resazurin reduction assay. Solvent served as negative control and 0.1% saponine was included as technical positive control (not shown). In general, little differences were observed between the two HepG2 cell models. While concentrations up to 100 µM had no effects (quinidine) or little effects (d-THP) on cell viability, both inhibitors displayed strong cytotoxicity at 500 µM (SI Fig. 3A). Therefore, 100 µM was selected as final concentration for the subsequent cytotoxicity studies with the different PAs.

**Fig. 1:**
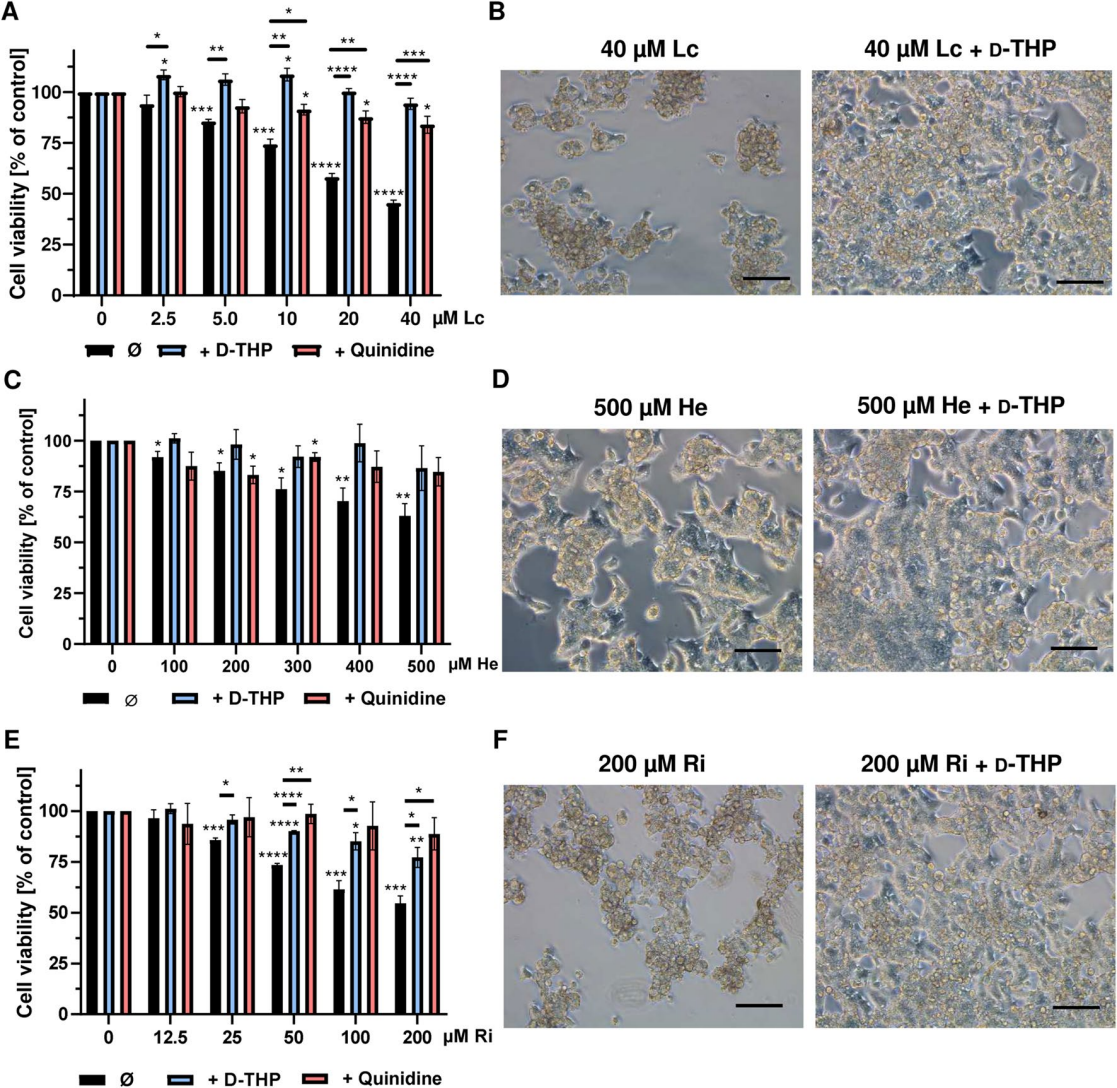
Pharmacological OCT1 inhibition and impact on PA-induced cytotoxicity in HepG2-CYP3A4 cells. **a**, **c**, **e** Viability of HepG2-CYP3A4 cells 24 h after incubation with increasing concentrations of lasiocarpine (**a**), heliotrine (**c**) and riddelliine (**e**) with or without OCT1-inhibitors. Solvent (0 µM) was used as negative control. Mean + SEM are shown for each incubation (n = 3, each measured as triplicates). Statistical analyses were performed using unpaired two-tailed Students t-test with respect to the negative control or the respective PA treatment as indicated. *P < 0.05, **P < 0.01, ***P < 0.001, ****P ≤ 0.0001. **b, d**,** f** Representative microscopic images of HepG2-CYP3A4 cells after 24 h treatment with different concentrations of heliotrine (**b**), lasiocarpine (**d**) and riddelliine (**f**) with or without d-THP. Scale bar represents 100 µm

We first determined the cytotoxicity of the open diester lasiocarpine in HepG2-CYP3A4 cells, which were incubated with increasing PA concentrations (0–40 µM) for 24 h in the absence or presence of the OCT inhibitors (100 µM each). As expected, lasiocarpine caused a concentration-dependent decrease in cell viability, with a reduction below 50% at a concentration of 40 µM (Fig. 1a). Strikingly, both d-THP and quinidine prevented the cytotoxic effects of lasiocarpine in HepG2-CYP3A4 cells (Fig. 1a), which was also observed by phase contrast microscopy (Fig. 1b and SI Fig. 4A). In wild-type HepG2 cells without CYP3A4 expression, lasiocarpine did not affect cell viability at all (SI Fig. 3B). We then studied the cytotoxicity of the monoester heliotrine with or without OCT inhibition. Cells were incubated with up to 500 µM heliotrine for 24 h, causing a drop in cell viability to 60% at the highest PA concentration (Fig. 1c). Both inhibitors rescued the cytotoxic effects of heliotrine, with d-THP being slightly more potent than quinidine, which was also visible by phase-contrast microscopy (Fig. 1c, d; SI Fig. 4B). Finally, the cyclic diester riddelliine was analyzed in HepG2-CYP3A4 cells in concentrations ranging from 0 to 200 µM. Cell viability was reduced by 50% at the top PA concentration, but almost completely restored to control levels upon quinidine co-treatment. d-THP was not as effective as quinidine, but nevertheless prevented the morphological changes induced by riddelliine (Fig. 1e, f; SI Fig. 4C). In summary, these results reveal an OCT1-mediated uptake of lasiocarpine, heliotrine and riddelliine into human liver cells.

### OCT1 and CYP3A4 as key determinants for PA-induced cytotoxicity

**Fig. 2:**
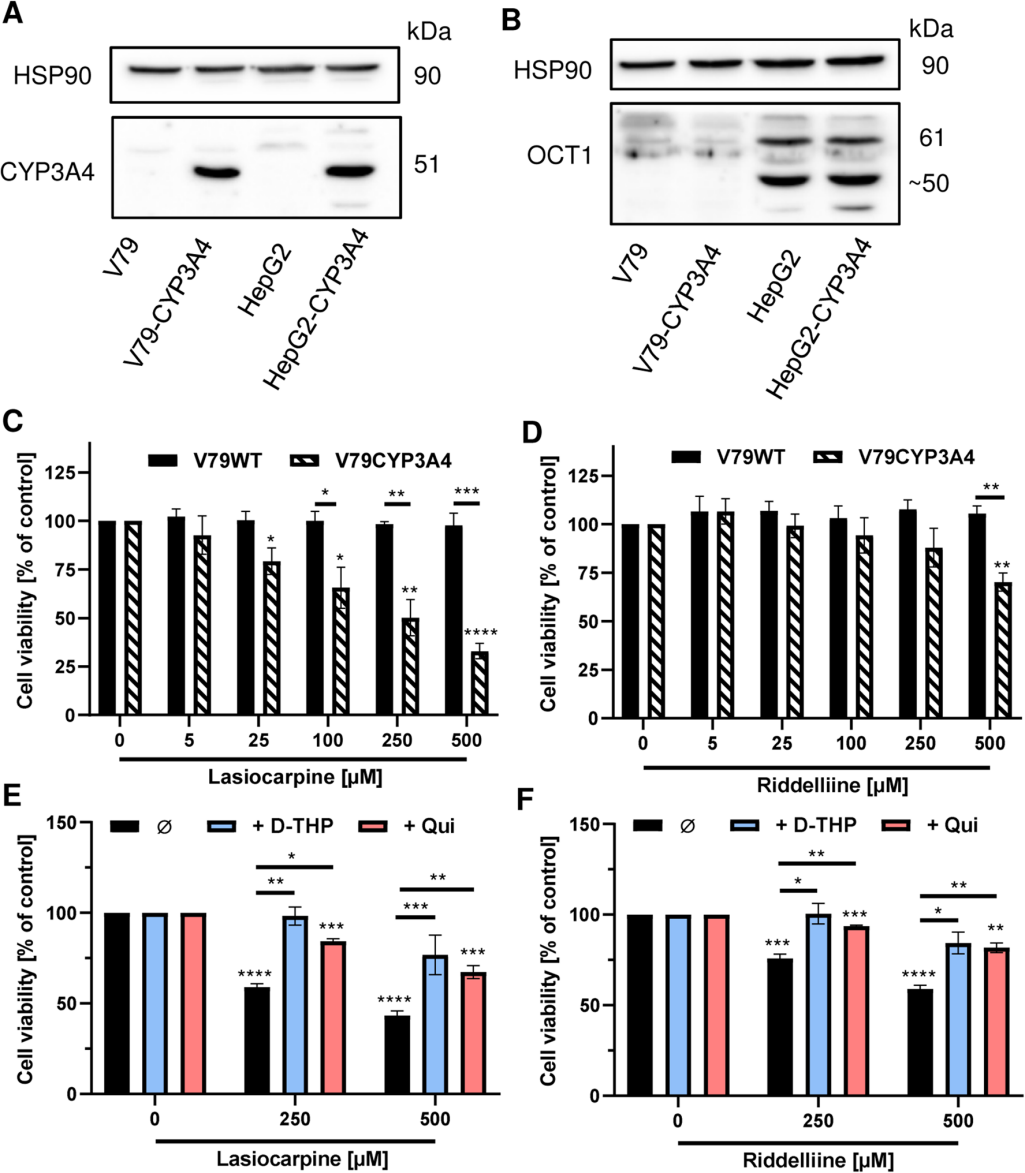
PA-triggered cytotoxicity in V79 and V79-CYP3A4 hamster cells and role of OCT1. **a**, **b** Analysis of CYP3A4 (**a**) and OCT1 (**b**) protein expression in V79, V79-CYP3A4, HepG2 and HepG2-CYP3A4 cells using SDS-PAGE and western blot detection. HSP90 served as loading control. A representative blot is shown. **c**, **d** Viability of V79 and V79-CYP3A4 cells 24 h after treatment with increasing concentrations of lasiocarpine (**c**) and riddelliine (**d**). Solvent (0 µM) was used as negative control. Mean + SEM are shown for each incubation (n = 3, each measured as triplicates). Statistical analyses were performed using unpaired two-tailed Students t-test with respect to the negative control or as indicated by bars. *P < 0.05, **P < 0.01, ***P < 0.001, ****P ≤ 0.0001. **e**, **f** Viability of V79-CYP3A4 cells 24 h after treatment with 250 and 500 µM lasiocarpine (**e**) or riddelliine (**f**) in the presence or absence of OCT1 inhibitors (quinidine and d-THP, 100 µM each). Solvent (0 µM) was used as negative control. Mean + SEM are shown for each incubation (n = 3, each measured as triplicates). Statistical analyses were performed using unpaired two-tailed Students t-test with respect to the negative control or as indicated by bars. *P < 0.05, **P < 0.01, ***P < 0.001, ****P < 0.0001

The role of OCT1 was further detailed in V79 Chinese hamster fibroblasts and genetically engineered V79 cells with human CYP3A4 expression. V79-CYP3A4 cells display a comparable CYP3A4 expression level as HepG2-CYP3A4 cells, which was demonstrated by western blot analysis (Fig. 2a). In contrast to that, OCT1 levels were much lower in both V79 and V79-CYP3A4 cells as compared to their HepG2 counterparts (Fig. 2b). We were thus interested how the strongly reduced OCT1 levels affect the cytotoxicity of the selected PAs. Lasiocarpine caused a concentration-dependent decrease of viability in V79-CYP3A4 cells, with a concentration of 250 µM reducing the viability by 50% as compared to control (Fig. 2c). A similar cytotoxic effect was already observed at a tenfold lower concentration in HepG2-CYP3A4 cells (see Fig. 1a). Riddelliine also exerted cytotoxicity in V79-CYP3A4 cells and decreased the viability to 70% at a concentration of 500 µM (Fig. 2d), whereas a comparable response was measured in HepG2-CYP3A4 cells upon incubation with only 50 µM riddelline (see Fig. 1e). Heliotrine displayed only little cytotoxicity in V79-CYP3A4 cells at concentrations of 250 µM and above (SI Fig. 5A), while it reduced the viability in HepG2-CYP3A4 cells to about 60% at a concentration of 500 µM (see Fig. 1c). It should also be noted here that neither lasiocarpine nor riddelline or heliotrine induced cytotoxicity in parental, metabolically incompetent V79 cells (Fig. 2c, d; SI Fig. 5A). As a next step, we tested whether OCT inhibition with d-THP or quinidine also protects V79-CYP3A4 cells against the PA-induced cytotoxicity. Our findings revealed that d-THP almost completely blocked the cytotoxic effects of lasiocarpine, riddelliine and heliotrine in V79-CYP3A4 cells (Fig. 2e, f; SI Fig. 5B). Quinidine also prevented the cytotoxicity of all three PAs, but was somewhat less active than d-THP. These results provided evidence that low OCT1 expression levels confer resistance towards the cytotoxic effects of PAs and that both OCT1 and CYP3A4 are required for PA toxicity.

### OCT1 inhibition prevents PA-mediated cytotoxicity in primary human hepatocytes

**Fig. 3:**
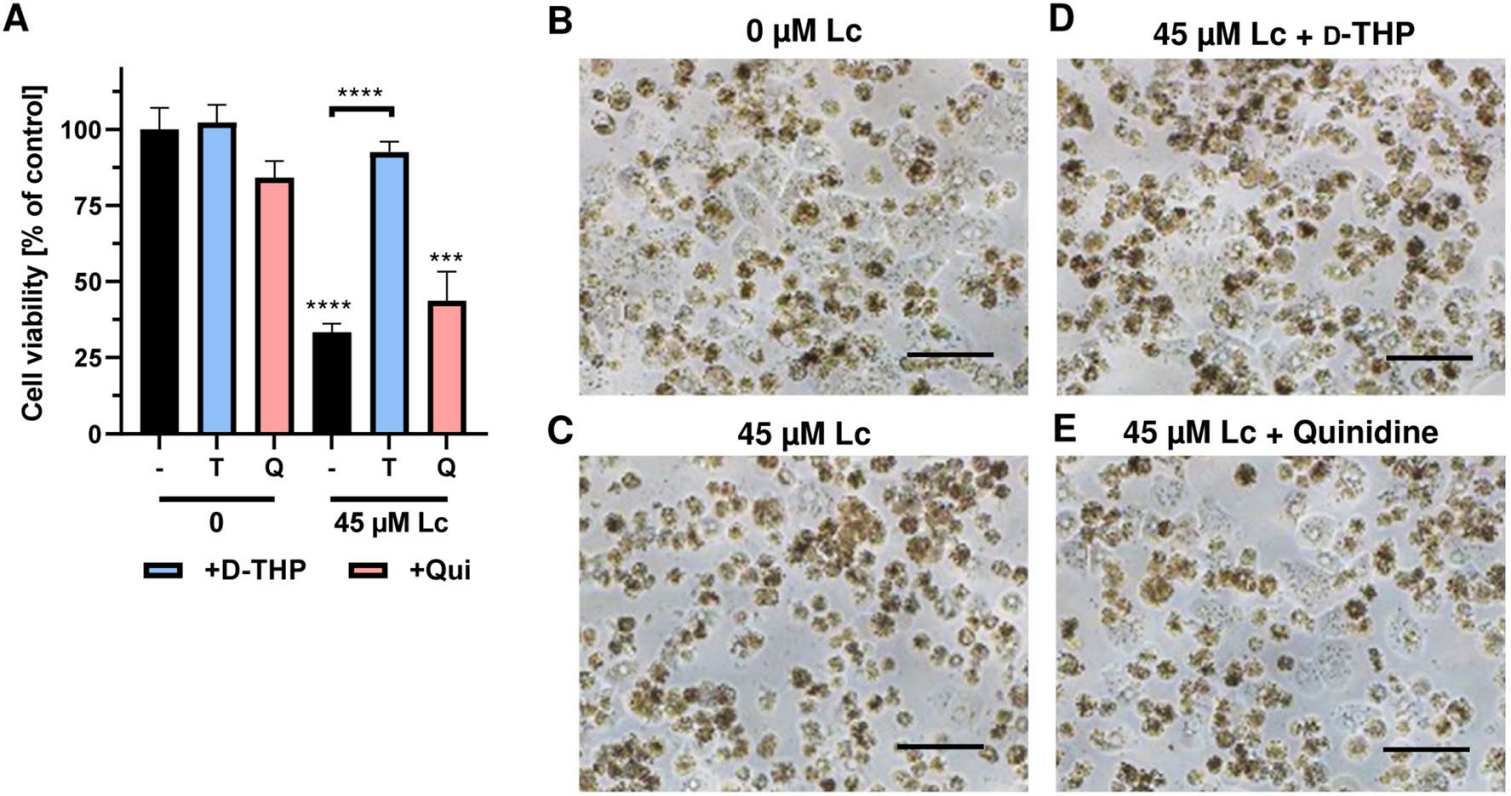
Lasiocarpine triggered cytotoxicity in primary human hepatocytes and impact of OCT1-mediated transport. **a** Viability of primary human hepatocytes 24 h after incubation with 45 µM lasiocarpine with or without the OCT1 inhibitors d-THP (T) and Quinidine (Q). Solvent (0 µM) was used as negative control. Mean + SEM are shown for each incubation (pooled hepatocytes from 5 donors, n = 2, each performed as triplicate). Statistical analyses were performed using unpaired two-tailed Students t-test with respect to the negative control or as indicated with a bar. ***P < 0.001, ****P < 0.0001. **b**–**e**: Representative microscopic images of primary human hepatocytes after 24 h incubation with lasiocarpine in the absence or presence of the OCT1 inhibitors. The scale bar represents 100 µm

Primary human hepatocytes (PHH) are the gold standard for in vitro toxicokinetic studies of the liver, since they display full metabolic competence and express a plethora of efflux and influx transporters (Fraczek et al. 2013; Ruoss et al. 2020). Up to date, no study is available that investigated the transport of PAs into PHHs. Based on our obtained results in HepG2-CYP3A4 as well as V79-CYP3A4 cells and our previous study in different human liver cell models (Haas et al. 2023), we selected lasiocarpine at a concentration of 45 µM for these experiments in PHH. Our results showed that lasiocarpine treatment decreased the viability of PHH to approximately 33% (Fig. 3a), which correlated with the morphological changes seen by phase contrast microscopy in lasiocarpine treated cells (Fig. 3b, c). d-THP itself had no effects, while quinidine caused a moderate reduction of viability in PHH (Fig. 3a). Interestingly, d-THP efficiently blocked the cytotoxic effects of lasiocarpine in PHH and restored viability almost to control levels (Fig. 3a). Quinidine also moderately increased the viability of lasiocarpine-treated cells, which was however not statistically significant as compared to the PA single exposure (Fig. 3a). The cytoprotective effects of d-THP and, to a minor degree, quinidine towards lasiocarpine were also visible using phase-contrast microscopy (Fig. 3d, e). The OCT1 inhibitors themselves did not induce changes in cell morphology (SI Fig. 6). Taken together, these findings in PHH corroborate the importance of OCT1 for hepatocellular PA uptake and toxicity.

### Pharmacological inhibition of OCT1-dependent transport abolishes the genotoxic effects and DNA damage response triggered by PAs in HepG2-CYP3A4 cells

**Fig. 4:**
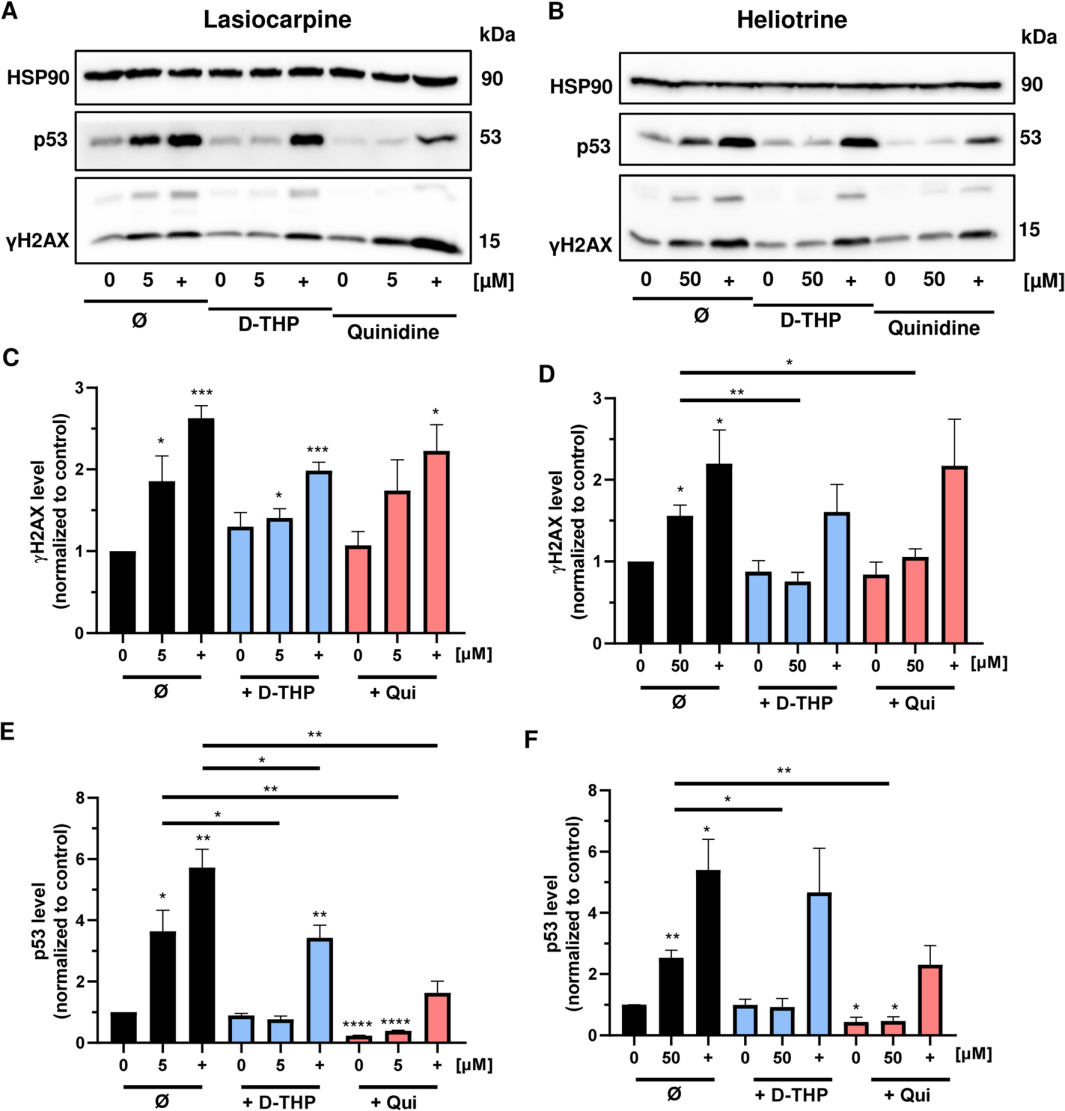
Impact of OCT1-inhibition on the genotoxicity of PAs in HepG2-CYP3A4 cells. **a**, **b** Representative western blots of γH2AX and p53 after 24 h treatment with lasiocarpine (**a**) and heliotrine (**b**). The genotoxic anticancer drug irinotecan was used as a positive control (+) and solvent as a negative control (0 µM). HSP90 served as loading control. **c**, **d** Densitometric evaluation of γH2AX after 24 h incubation with lasiocarpine (**c**) and heliotrine (**d**) in HepG2-CYP3A4 cells. HSP90 served as loading control. γH2AX level relative to the loading control and normalized versus the negative control. Mean + SEM for three independent experiments (n = 3). Statistical analyses were performed using unpaired two-tailed Students t-test with respect to the negative control or as indicated with a bar. *P < 0.05, **P < 0.01, ***P < 0.001. **e**, **f** Densitometric evaluation of p53 after 24 h incubation with lasiocarpine (**e**) and heliotrine (**f**) in HepG2-CYP3A4 cells. HSP90 served as loading control. p53 level relative to the loading control and normalized versus the negative control. Mean + SEM for three independent experiments (n = 3). Statistical analyses were performed using unpaired two-tailed Students t-test with respect to the negative control or as indicated with a bar. *P < 0.05, **P < 0.01, ****P < 0.0001

We were then interested whether the reduced cytotoxicity following OCT inhibition is related to an attenuated genotoxicity. Thus, we assessed the genotoxicity of the selected PAs (heliotrine, lasiocarpine and riddelliine) in HepG2-CYP3A4 cells using the well-established DNA damage markers γH2AX and p53 (Fahrer et al. 2015; Nikolova et al. 2014) in the presence or absence of the OCT inhibitors. HepG2-CYP3A4 cells were exposed to 5 µM lasiocarpine, 12.5 µM riddelliine and 50 µM heliotrine for 24 h. The lower PA concentrations were chosen to avoid overt cytotoxicity. The genotoxic anticancer drug and topoisomerase I inhibitor irinotecan was included as positive control (10 µM), since it was previously described as an OCT substrate (Chen et al. 2016; Hucke and Ciarimboli 2016). All tested PAs caused substantial γH2AX formation and p53 accumulation in HepG2-CYP3A4 cells (Fig. 4a–f; SI Fig. 7A–C). d-THP strongly attenuated γH2AX levels and reduced p53 levels to control levels despite PA exposure (Fig. 4a–f; SI Fig. 7A–C). Likewise, quinidine prevented the formation of both genotoxicity markers, although with differential efficacy. While p53 levels were even reduced below baseline, the effects on γH2AX induction were not as pronounced, at least in the case of lasiocarpine (Fig. 4a, c). As expected, irinotecan treatment caused strong genotoxic effects in HepG2-CYP3A4 cells, which were attenuated by d-THP (Fig. 4a–f; SI Fig. 7A–C). Quinidine, in turn, did not or hardly prevent γH2AX formation upon irinotecan exposure, but suppressed its p53 induction (Fig. 4a–f; SI Fig. 7A–C).

**Fig. 5:**
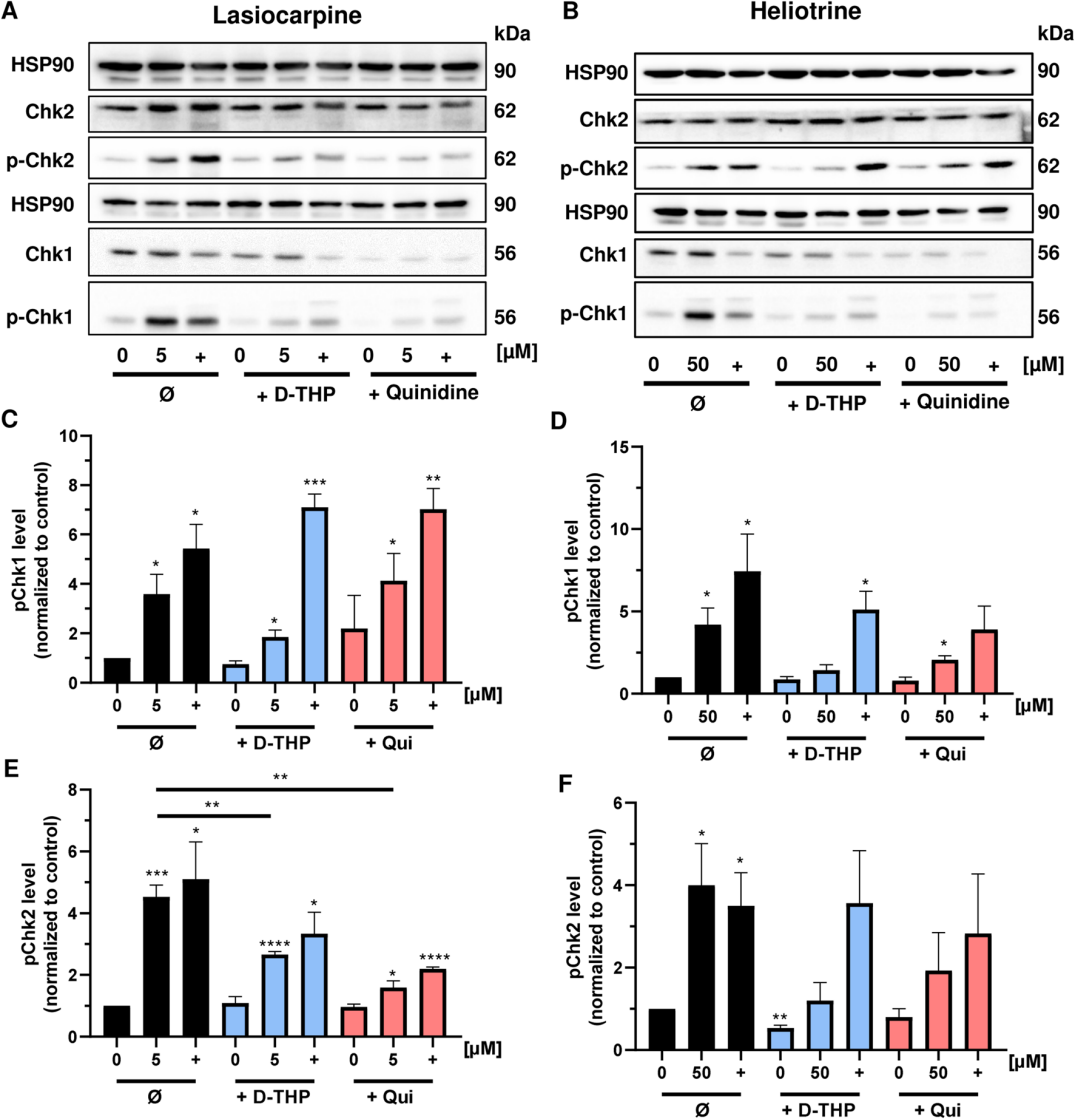
OCT1-inhibition and PA-triggered DNA damage response (DDR). **a**, **b** Representative western blots of (phosphorylated) CHK1 and CHK2 as downstream targets of the apical DDR kinases ATR and ATM after 24 h treatment with lasiocarpine (**a**) and heliotrine (**b**). The genotoxic anticancer drug irinotecan was used as a positive control (+) and solvent as a negative control (0 µM). HSP90 served as loading control. **c**, **d** Densitometric evaluations of p-Chk1 (S345) after 24 h incubation with lasiocarpine (**c**) and heliotrine (**d**) in HepG2-CYP3A4 cells. Unphosphorylated CHK1 served as loading control. pCHK1 level relative to the loading control and normalized versus the negative control. Mean + SEM for three independent experiments (n = 3). Statistical analyses were performed using unpaired two-tailed Students t-test with respect to the negative control or as indicated with a bar. *P < 0.05, **P < 0.01, ***P < 0.001. **e**, **f** Densitometric evaluations of pChk2 (Thr68) after 24 h incubation with lasiocarpine (**e**) and heliotrine (**f**) in HepG2-CYP3A4 cells. Unphosphorylated CHK2 served as loading control. pCHK2 level relative to the loading control and normalized versus the negative control. Mean + SEM for three independent experiments (n = 3). Statistical analyses were performed using unpaired two-tailed Students t-test with respect to the negative control or as indicated with a bar. *P < 0.05, **P < 0.01, ***P < 0.001, ****P < 0.0001

Finally, we analyzed the DNA damage response (DDR) triggered by the three selected PAs in HepG2-CYP3A4 cells and studied the impact of OCT inhibition. The DDR is coordinated by apical DDR kinases including ATM and ATR, which phosphorylate a plethora of downstream substrates such as the checkpoint kinases CHK1 and CHK2 (Marechal and Zou 2013). While CHK1 is primarily phosphorylated by ATR in response to DNA replication stress, CHK2 is mainly phosphorylated by ATM following DNA double-strand breaks (Rundle et al. 2017; Shiloh and Ziv 2013). First, HepG2-CYP3A4 cells were exposed to the three structurally diverse PAs lasiocarpine, heliotrine and riddelliine for 24 h as described above. Western Blot analysis revealed a pronounced increase in pCHK1 and pCHK2 levels upon exposure to all PAs, whereas the levels of total CHK1 and CHK2 were unchanged (Fig. 5a, b; SI Fig. 8A). Co-treatment of cells with the OCT inhibitors d-THP and quinidine reduced phosphorylation of CHK1 and CHK2 upon PA exposure (Fig. 5a–f; SI Fig. 8A–C), which is in line with the reduced genotoxicity of PAs upon OCT inhibition (see Fig. 4). The anticancer drug irinotecan also caused genotoxicity (see Fig. 4a, b) as well as CHK1 and CHK2 phosphorylation (Fig. 5a, b; SI Fig. 8A). However, the OCT inhibitors displayed little or no effects on these DDR markers induced by irinotecan. In summary, our findings show that OCT1-dependent uptake of PAs is required for PA triggered genotoxicity and activation of the ATM/ATR-driven DDR, which precedes the cytotoxic effects.

## Discussion

In the present work, we investigated the relevance of OCT1 for the uptake of structurally diverse PAs in metabolically competent human HepG2-CYP3A4 cells, Chinese hamster V79-CYP3A4 cells and PHH. HepG2-CYP3A4 cells were revealed as a very useful model in our previous study, since the genotoxic and cytotoxic potency of structurally diverse PAs was in very good agreement with that obtained in PHH as gold standard (Haas et al. 2023). Furthermore, HepG2 cells were reported to display *OCT1* gene expression, although the expression levels were lower than those in PHH (Herzog et al. 2016; Rodrigues et al. 2009). Importantly, OCT1 expression was confirmed on the protein level in HepG2-CYP3A4 and wildtype HepG2 cells as shown above, thus representing a valuable model to study OCT1-mediated uptake of PAs. Here, we made use of the established pharmacological OCT1 inhibitors quinidine and d-THP (Ingoglia et al. 2015; Tu et al. 2013), which were instrumental to study the uptake of other PAs (see below). However, it should be noted that both quinidine and d-THP undergo CYP-mediated phase I metabolism. Quinidine is a known substrate of CYP3A4, which catalyzes its 3-hydroxylation (Nielsen et al. 1999). d-THP was reported to be a substrate for rat CYP3A1/2, the orthologue of human CYP3A4 (Zhao et al. 2012). Thus, it might be possible that the tested PAs and the inhibitors compete for CYP3A4, thereby decreasing the metabolic activation rate of PAs. Importantly, inhibitory effects of d-THP were only observed for CYP2D6 and CYP1A2 activities (Li et al. 2015).

First, we were able to demonstrate that pharmacological OCT1 inhibition by either d-THP or quinidine rescued the cytotoxic effects of all three PAs tested, i.e. lasiocarpine, heliotrine and riddelliine, in HepG2-CYP3A4 cells. Our results for riddelliine extend the previous findings obtained for the other cyclic PA diesters monocrotaline, retrorsine and senecione, which were shown to be taken up into kidney MDCK-hOCT1 cells, primary rat hepatocytes as well as HepaRG cells in a OCT1-dependent manner (Enge et al. 2021; Tu et al. 2013, 2014). Furthermore, our data showed for the first time that also open-chained PA diesters as well as PA monoesters are substrates for OCT1. These findings were confirmed with another set of experiments in V79-CYP3A4 cells, in which OCT1 inhibition conferred resistance towards the cytotoxic effects of the three PAs tested. Interestingly, the cytotoxicity observed in V79-CYP3A4 cells was generally lower than that detected in HepG2-CYP3A4 cells, although both cell lines displayed similar CYP3A4 protein levels. However, OCT1 expression was revealed to be much higher in HepG2-CYP3A4 cells, which very likely explains their increased sensitivity towards PAs and further emphasizes the relevance of OCT1 for PA-triggered toxicity. Apart from that, V79 Chinese hamster fibroblasts are known to harbor a mutated and non-functional p53 (Chaung et al. 1997), whereas HepG2 cells display wild-type p53 (Müller et al. 1997).

Due to its high cytotoxic potency, lasiocarpine was selected for further analysis in PHH as gold standard for toxikokinetic studies. Consistent with the results in HepG2-CYP3A4 and V79-CYP3A4 cells, d-THP almost completely inhibited the cytotoxic effects of lasiocarpine, while quinidine only moderately increased the cell viability. The reduced effectiveness of quinidine might be attributable to its own toxicity and/or the differences in the metabolic competence of PHH vs. HepG2-CYP3A4 cells.

Then we addressed the question whether the reduced cytotoxicity upon OCT1 inhibition is associated with an attenuated genotoxicity. Our results provided evidence that OCT1 inhibition also prevents the genotoxic effects of all three selected PAs as attested by reduced DNA damage levels (γH2AX and p53). However, quinidine only partially blocked γH2AX formation triggered by lasiocarpine, whereas it strongly attenuated γH2AX formation caused by heliotrine and riddelliine. This finding might indicate that low intracellular levels of lasiocarpine are sufficient to cause substantial γH2AX formation, bearing in mind that this is an open-chained PA diester with a higher genotoxic potency than the cyclic PA diester riddelliine and the PA monoester heliotrine (Haas et al. 2023). Furthermore, we analyzed the DNA damage response markers CHK1 and CHK2. These checkpoint kinases are phosphorylated by apical DDR kinases, namely ATM, ATR and/or DNA-PK, in response to diverse genotoxic stimuli (Marechal and Zou 2013). First evidence for a DDR activation stems from a 28 day rat feeding study with different PAs. Transcriptomic analysis of liver tissue showed an enrichment of DDR pathways such as p53 and ATM signaling (Ebmeyer et al. 2020), which was further substantiated with a transcriptomics study performed in HepG2-CYP3A4 cells (Abdelfatah et al. 2021). Intriguingly, we were able to show that all three PAs (50 µM heliotrine, 5 µM lasiocarpine and 12.5 µM riddelliine) significantly increased both CHK1 and CHK2 phosphorylation in HepG2-CYP3A4 cells after 24 h. This is consistent with experiments performed in genetically engineered TK6 cells with human CYP3A4 expression, which displayed CHK1 and CHK2 phosphorylation upon exposure to lasiocarpine (5 µM) and riddelliine (20 µM) for 24 h. Moreover, our data revealed that OCT1 inhibition (d-THP  > quinidine) blocked PA triggered DDR activation as evidenced by reduced pCHK1 and pCHK2 levels.

In conclusion, we demonstrated that PAs independent of their degree of esterification are substrates for OCT1-mediated uptake into human liver cells. We further provided evidence that OCT1 inhibition prevents PA triggered genotoxicity, DDR activation and subsequent cytotoxicity, highlighting the crucial role of OCT1. Our results have also implications for the toxicity of PAs in vivo, since the cell- and tissue-dependent expression of OCTs have a major effect on their susceptibility towards the detrimental effects of PAs as demonstrated herein. Finally, the better understanding of the involved hepatocellular uptake mechanisms might also open an avenue for the protection of (liver) cells in case of an acute intoxication by using i.v. administered OCT1 inhibitors.

## Material and methods

### Cell culture and treatment

V79 Chinese hamster cells and genetically engineered V79 cells with stable expression of human CYP3A4 (Ebmeyer et al. 2019) were grown in Dulbecco`s modified Eagle`s Medium (DMEM) high glucose supplemented with 5% fetal calf serum (FCS) and 1% penicillin/streptomycin (P/S). HepG2 cells were obtained from DSMZ (Braunschweig, Germany) and HepG2-CYP3A4 cells were generated as previously described (Herzog et al. 2015). HepG2-CYP3A4 cells were maintained in DMEM high glucose supplemented with 10% FCS, 1% P/S and 3 µg/ml blasticidin S hydrochloride (Carl Roth, Karlsruhe, Germany). All cell lines were cultured at 37 °C in humidified atmosphere of 5% CO_2_. Cell culture medium and supplements were obtained from Gibco Life Technologies (Darmstadt, Germany) and Pan Biotech (Aidenbach, Germany). All cell lines were mycoplasma negative, as demonstrated by routine PCR testing using Venor^®^GeM OneStep (Berlin, Germany). Cryopreserved PHH pooled from five Caucasian donors were from Thermo Fisher Scientific (Massachusetts, USA) and were maintained as described recently (Haas et al. 2023). After attachment of PHH to collagen type I coated plates, the plating medium was replaced with incubation medium containing the test compounds and cells were incubated for 24 h. Collagen type I was obtained from Corning (New York, USA) and prepared as sterile-filtered 50 µg/ mL stock solution in 0.8 M acetic acid for coating.

### Compounds and cell treatment with OCT1 inhibitors

The PAs used (heliotrine, lasiocarpine, riddelliine) were of highest purity and were from Phytolab (Vestenbergsgreuth, Germany). All PAs were dissolved in DMSO to prepare stock solutions (50–150 mM), which were stored at − 20 °C. Two OCT1 inhibitors, namely d-tetrahydropalmatine (d-THP) and quinidine, were purchased from Hycultec (Beutelsbach, Germany) and dissolved in DMSO to obtain 100 mM stock solutions stored at − 20 °C. Stock solutions were diluted in cell culture medium to reach final concentrations in the experiments as indicated (typically 100 µM). For the inhibitor studies, cells were pre-incubated for 1.5 h with 100 µM d-THP or quinidine. The medium was then aspirated and fresh medium was added, which contained the PAs under investigation with or without 100 µM of the respective OCT1-inhibitor for 24 h. Cells were then analyzed as described below.

### Assessment of cell viability

HepG2 and HepG2-CYP3A4 cells (45,000 cells/ well) as well as V79 and V79-CYP3A4 cells (5,000 cells/ well) were seeded on 96-well plates, grown overnight and incubated with increasing PA concentrations without or with 100 µM of each OCT1-inhibitor. PHH were seeded on collagen type I coated 96-well plates at density of 62,500 cells/well and treated as described above. Saponine (0.1%) served as positive control and solvent (DMSO) as negative control. Cell viability was determined using the resazurin reduction assay as described previously (Carlsson et al. 2022). After 24 h, cells were washed with PBS and incubated for 1 h with DMEM low glucose (-FCS; -P/S) or William’s E Medium (1X) supplemented with 10% 440 µM resazurin-NaCl-Pi-solution (0.1% dimethylformamide; 1.1 mM KH_2_PO_4_, 154 mM NaCl, 3.7 mM Na_2_HPO_4_) at 37 °C in humidified atmosphere of 5% CO_2_. Cell viability was determined using a microplate reader (Spark, Tecan) with 544 nm for excitation and 590 nm for emission. In addition, phase-contrast microscopy was performed using a Leica microscope (HI PLAN I 20x/0.30 PH1 or HI PLAN I 10x/0.22 PH1) equipped with Leica MC170 HD camera.

### SDS-PAGE and western blot analysis

HepG2-CYP3A4 cells (500,000 cells/plate) were grown on 3.5 cm plates, incubated with increasing PA concentrations for 24 h and directly harvested with 1 × Laemmli loading buffer as described (Fahrer et al. 2014). Samples were then subject to SDS-PAGE and western blot analysis as reported (Fahrer et al. 2013). Briefly, proteins were separated by SDS-PAGE and transferred onto a nitrocellulose membrane (PerkinElmer, Rodgau, Germany) with wet blot technique. The membrane was blocked with 5% nonfat dry milk or 5% bovine serum albumine (BSA) in Tris-buffered saline (TBS)/ 0.1% Tween-20 (TBS-T) for 1 h at RT. As a next step, the membranes were incubated with the primary antibody overnight at 4 °C. The membranes were washed three times with TBS-T and incubated with appropriate secondary antibodies conjugated with horseradish peroxidase (HRP) for 1 h at RT. After additional washing steps, the membranes were incubated with Western Lighting^®^ Plus-ECL (PerkinElmer, Rodgau, Germany) and proteins were visualized with a c300 chemiluminescence imager (Azure biosystems, Dublin, CA, USA). The primary antibodies directed against CYP3A4 (HL3, sc-53580), p53 (DO-1, sc-126) and HSP90α/β (sc-13119) were obtained from Santa Cruz Biotechnology (Heidelberg, Germany). The primary antibody raised against γH2AX (phosphor S139, ab81299) was from Abcam (Cambridge, UK). The primary antibodies p-CHK1 (phosphor S345, #2348) and p-CHK2 (phosphor Thr68, #2197) as well as CHK1 (#2360) and CHK2 (#2662) were obtained from Cell Signaling Technology (Danvers, Massachusetts, USA). The primary antibody directed against OCT-1/SLC22A1 (2C5, NBP1-51684) was obtained from Novus Biologicals LLC (Centennial, Colorado, USA). HRP-conjugated secondary antibodies conjugated were purchased from Santa Cruz Biotechnology (sc-516102, Heidelberg, Germany) and Cell Signaling Technology (#7074, Danvers, Massachusetts, USA).

### Immunofluorescence and confocal microscopy

HepG2 and HepG2-CYP3A4 cells were seeded on cover slips in 3.5-cm dishes (4 × 10^5^ per dish) and allowed to grow for 24 h. Immunofluorescence staining and confocal microscopy were essentially performed as reported (Mimmler et al. 2016). To this end, cells were rinsed with PBS and fixed with 4% paraformaldehyde (PFA) for 15 min at RT. The PFA solution was discarded and cells were fixed additionally with ice-cold methanol for 10 min at − 20 °C. Thereafter, cells were washed three times with PBS and unspecific binding sites were blocked with 5%-BSA in PBS/0.3%-Triton-X100 in PBS. The samples were then incubated with a primary antibody against CYP3A4 (1:200 in 0.3%-Triton-X100 in PBS; HL-3, sc-53580, Santa Cruz Biotechnology, Heidelberg, Germany) overnight at 4 °C. The cells were washed with PBS and PBS/0.4 M NaCl before incubation with a secondary antibody labeled with Alexa Fluor 488 (1:400 in 5%-BSA in PBS/ 0.3%-Triton-X100; Life Technologies, Darmstadt, Germany) for 1.5 h at room temperature. Cells were then washed again as described above and embedded with Vectashield-DAPI (Vector Labs, Burlingame, CA, USA). The samples were analyzed with a Zeiss Axio Observer 7 microscope equipped with 63-oil-objective (plan-apochromat 63x/1.40 DIC M27) and the LSM 900 confocal laser scanner. The images were acquired and processed with Zen Software 3.4 (Carl Zeiss Microscopy, Jena, Germany).

### Statistical analysis

All experiments were performed independently at least three times, except otherwise stated. Results are presented as means + standard error of the means (SEM) from representative experiments. Statistics were carried out by Graphpad Prism software (Version 9). Statistical significance was defined as P < 0.05 and statistical analyses were performed using unpaired two-tailed Students t-test with respect to the negative control.

### Supplementary Information

Below is the link to the electronic supplementary material.

Supplementary file1 (DOCX 4390 KB)

## Data Availability

The datasets generated and analyzed during this study were included in the manuscript and the supplementary information. They are also available from the corresponding author upon reasonable request.
