# Supplementary material for: OCT1-dependent uptake of structurally diverse pyrrolizidine alkaloids in human liver cells is crucial for their genotoxic and cytotoxic effects
Source: Arch Toxicol. 2023 Sep 7;97(12):3259–71. doi: 10.1007/s00204-023-03591-4 (PMC10567918; doi:10.1007/s00204-023-03591-4)
Supplement: Supplementary file 1 — Supplementary file1 (DOCX 4390 KB) [file 204_2023_3591_MOESM1_ESM.docx]

**SI Fig. 1**

**
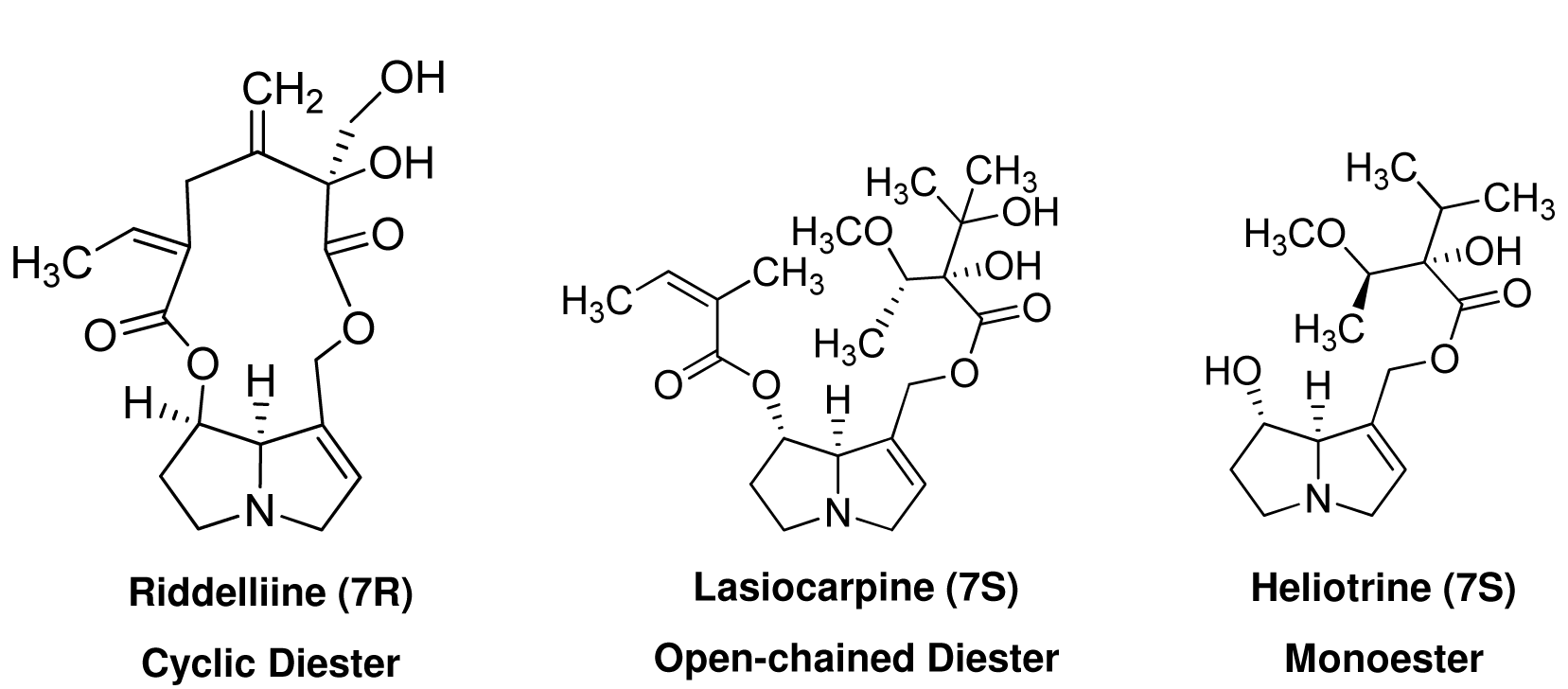
**

**SI Fig. 1: Chemical structures of the three selected PAs, which differ in their degree and type of esterification.**

**SI Fig. 2**

**
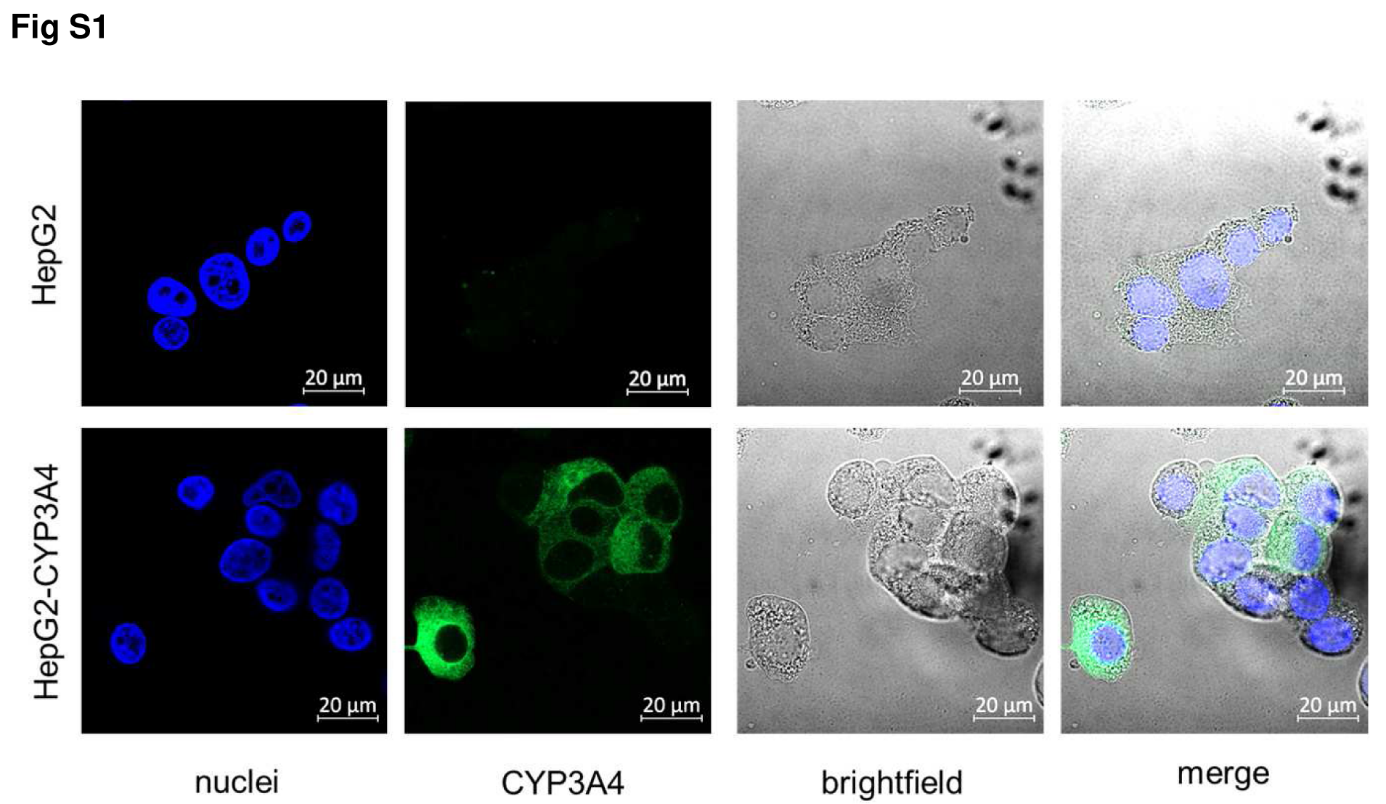
**

**SI Fig. 2: Expression of CYP3A4 in HepG2 and HepG2-CYP3A4 cells.** Immunostaining of CYP3A4 in wild-type HepG2 cells and genetically engineered HepG2-CYP3A4 cells. Nuclei were visualized with DAPI staining and cell morphology was assessed using brightfield. Representative confocal images are shown. Scale bar indicates 20 µm.

**SI Fig. 3**


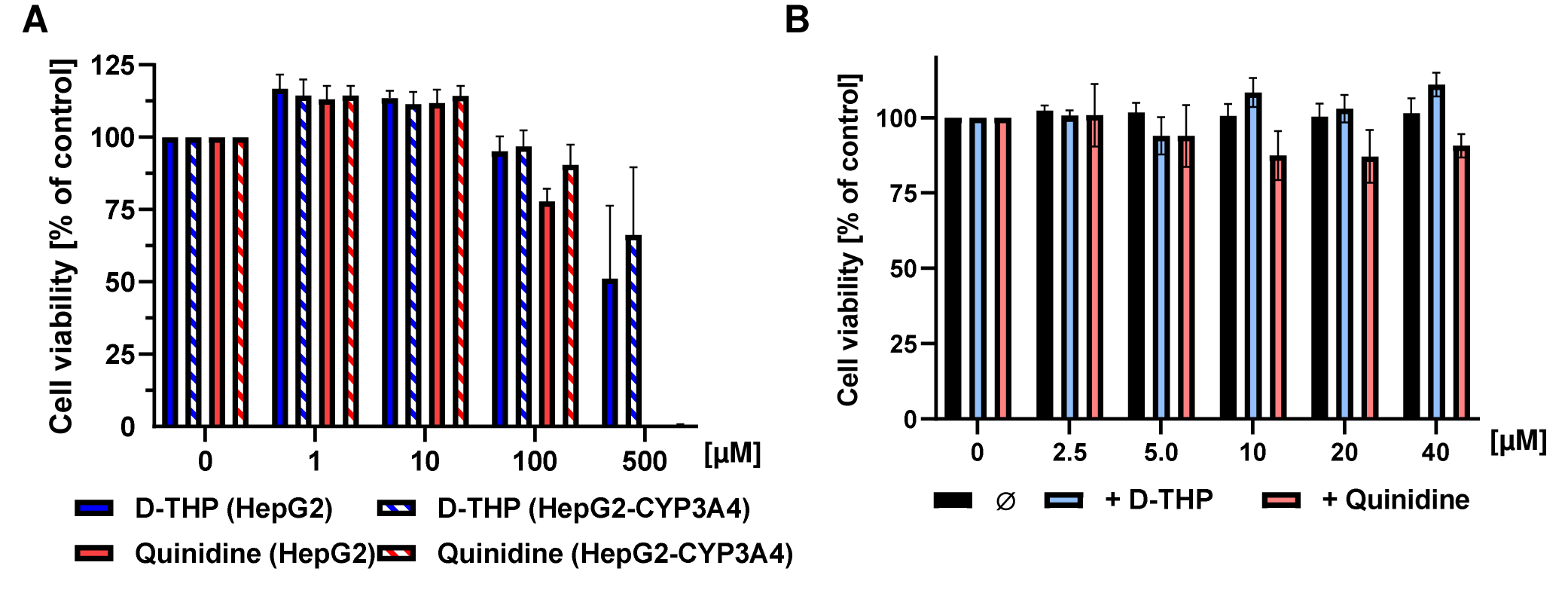


**SI Fig. 3: Cytotoxicity of pharmacological OCT1-inhibitors in HepG2 and HepG2-CYP3A4 cells and PA-mediated cytotoxicity in HepG2 cells with or without OCT1-inhibitors. A** Viability of HepG2 and HepG2-CYP3A4 cells 24 h after treatment with increasing concentrations of the OCT1 inhibitors D-THP and quinidine. **B** Viability of HepG2 cells 24 h after incubation with increasing concentrations of lasiocarpine with or without OCT1 inhibitors. Solvent (0 µM) was included as negative control. Mean + SEM are shown for each incubation (n=3, each measured as triplicates).

**SI Fig. 4**

**
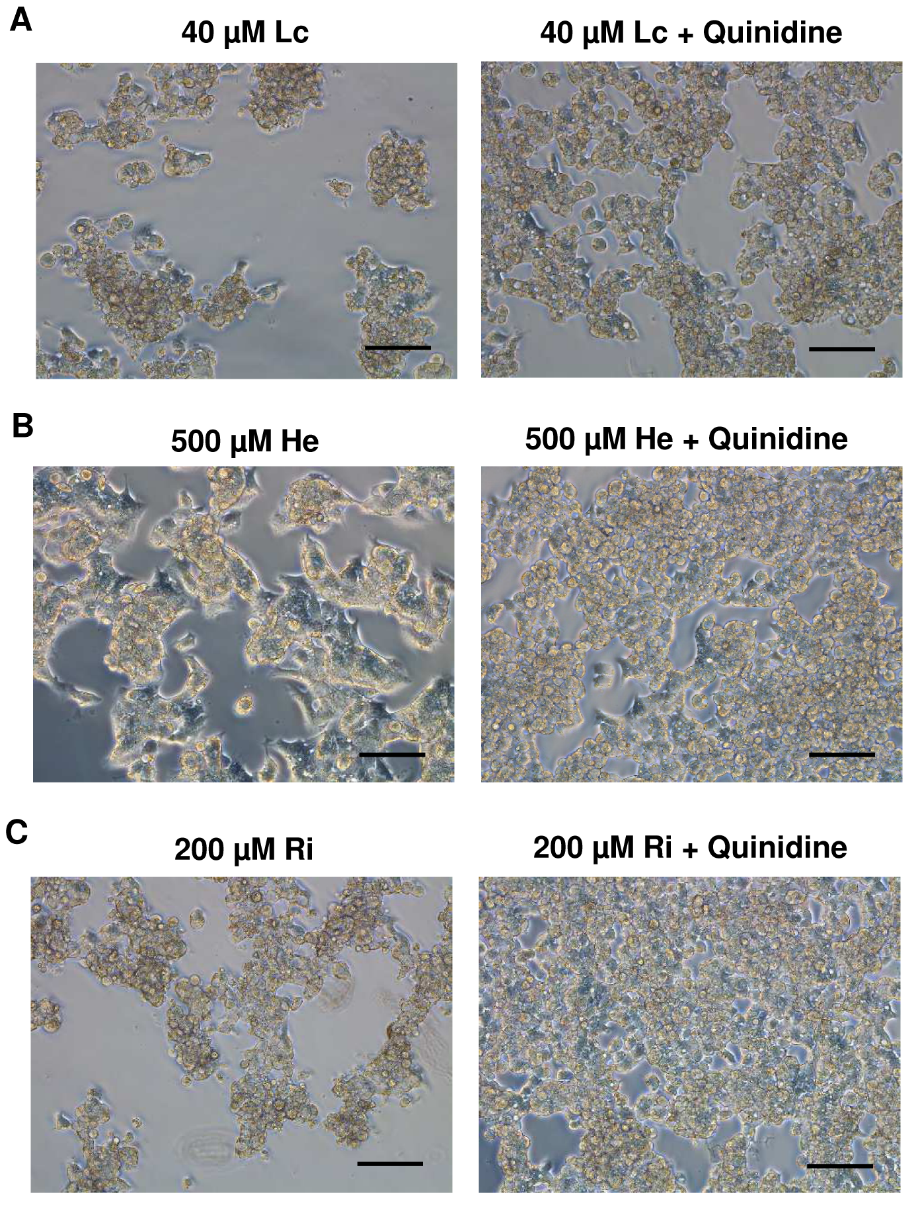
**

**SI Fig. 4: Pharmacological OCT1 inhibition and impact on PA-induced cytotoxicity in HepG2-CYP3A4 cells. A-C** Representative microscopic images of HepG2-CYP3A4 cells after 24 h treatment with lasiocarpine (A) heliotrine (B) and riddelliine (C) in the absence or presence of quinidine as OCT1 inhibitor. Scale bar represents 100 µm.

**SI Fig. 5**

**
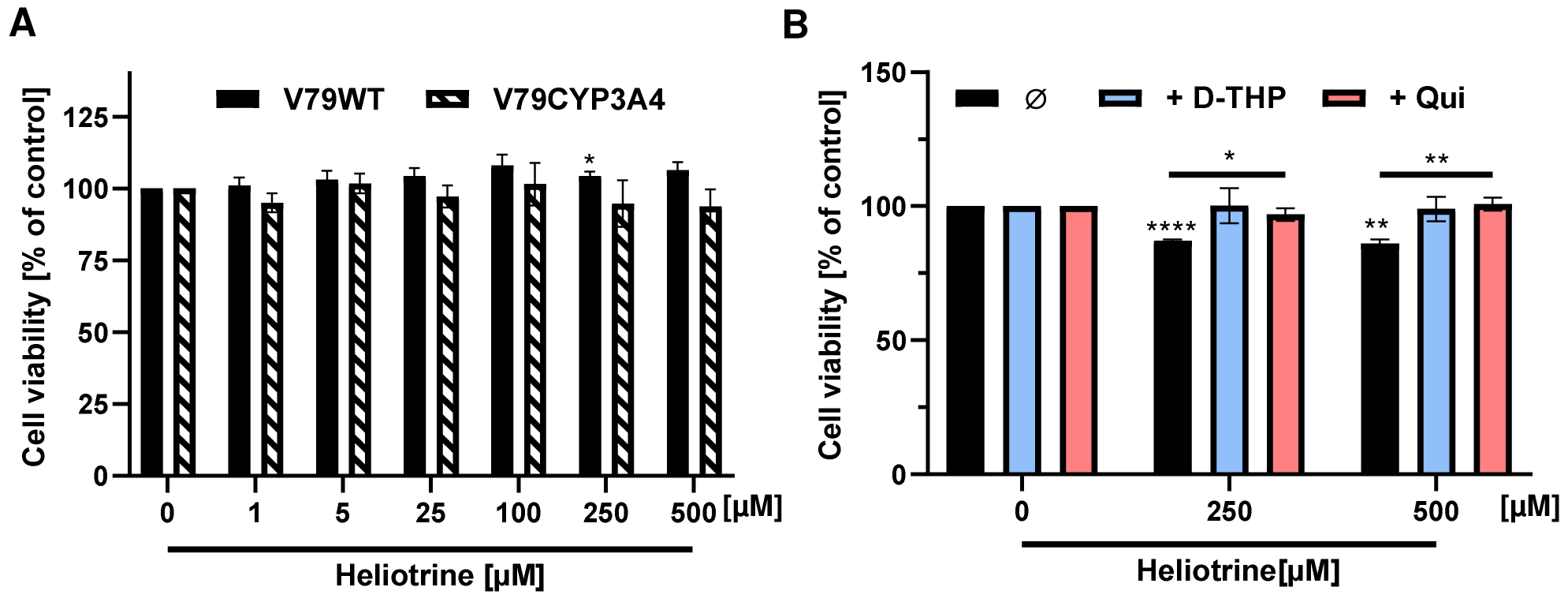
**

**SI Fig. 5: Cytotoxicity of heliotrine in V79 and V79-CYP3A4 cells and impact of OCT1-inhibition. A** Viability of V79 and V79-CYP3A4 cells 24h after treatment with increasing concentrations of heliotrine. Solvent (0 µM) was used as negative control. Mean + SEM for each incubation (n≥3, each measured as triplicates). Statistical analyses were performed using unpaired two-tailed Students t-test with respect to the negative control. *P<0.05. **B** Viability of V79-CYP3A4 cells 24 h after incubation with 250 and 500 µM heliotrine in the absence or presence of the OCT1-inhibitors D-THP and quinidine (100 µM each). Solvent (0 µM) was used as negative control. Mean + SEM for each incubation (n=3, each measured as triplicates). Statistical analyses were performed using unpaired two-tailed Students t-test with respect to the negative control or as indicated with a bar. *P<0.05, **P<0.01, ****P<0.0001.

**SI Fig. 6**

**
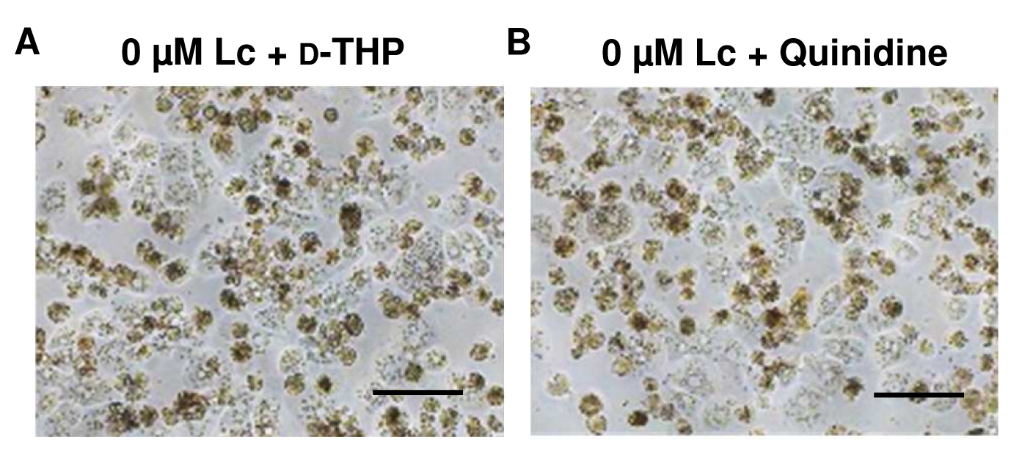
**

**SI Fig. 6: Impact of OCT1-inhibitiors on morphology of primary human hepatocytes. A** and **B** Representative microscopic images of primary human hepatocytes after 24 h incubation with solvent (0 µM) in the presence of the OCT1 inhibitors D-THP and Quinidine, respectively. The scale bar represents 100 µm.

**SI Fig. 7**

**
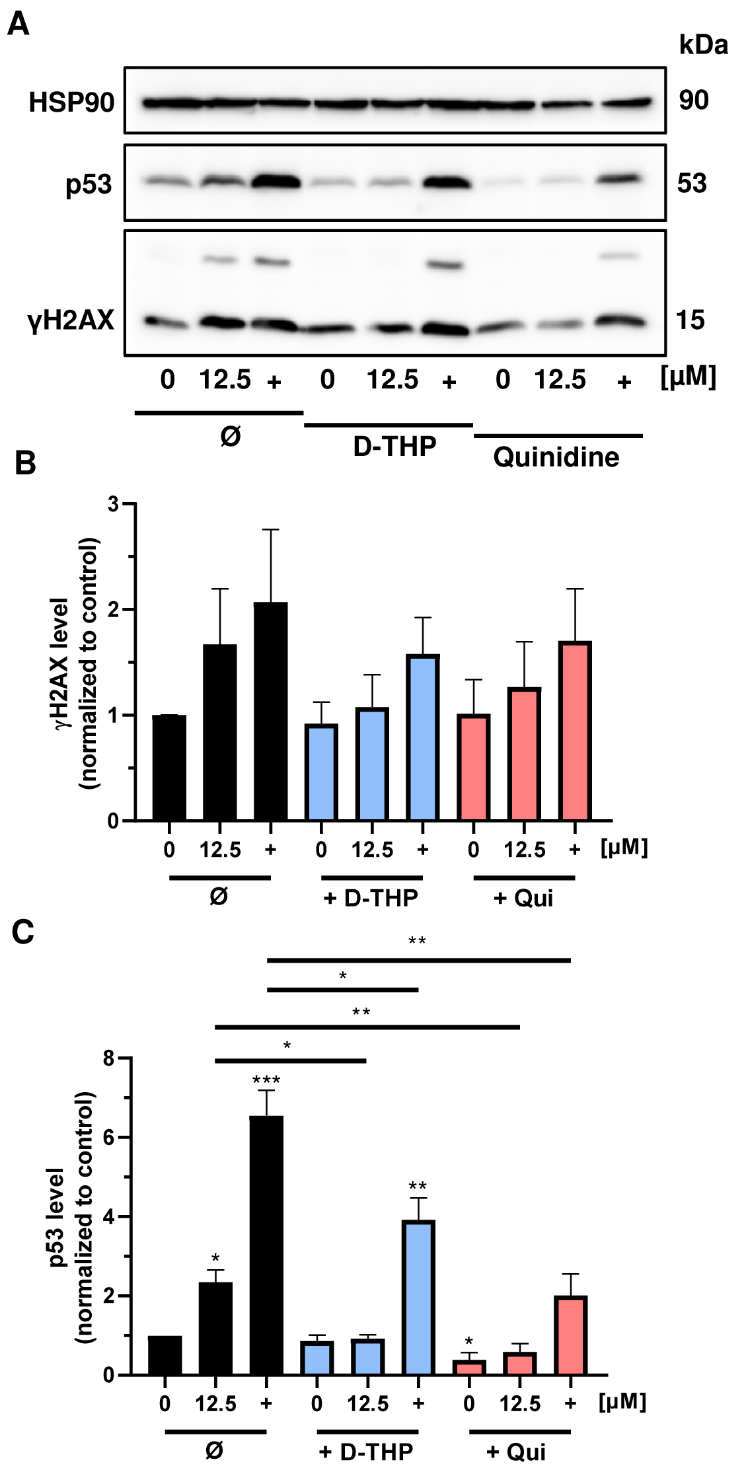
**

**SI Fig. 7: Impact of OCT1-inhibition on the genotoxicity of riddelliine in HepG2-CYP3A4 cells. A** Representative western blots of γH2AX and p53 after 24 h treatment with riddelliine. The genotoxic anticancer drug irinotecan was used as a positive control (+) and solvent as a negative control (0 µM). HSP90 served as loading control. **B** and **C**: Densitometric evaluation of γH2AX (B) and p53 (C) after 24 h incubation with lasiocarpine (C) and heliotrine (D) in HepG2-CYP3A4 cells. HSP90 served as loading control. γH2AX level and p53 level relative to the loading control and normalized versus the negative control. Mean + SEM for three independent experiments (n=3). Statistical analyses were performed using unpaired two-tailed Students t-test with respect to the negative control or as indicated with a bar. *P<0.05, **P<0.01, ***P<0.001, ****P<0.0001.

**SI Fig. 8**

**
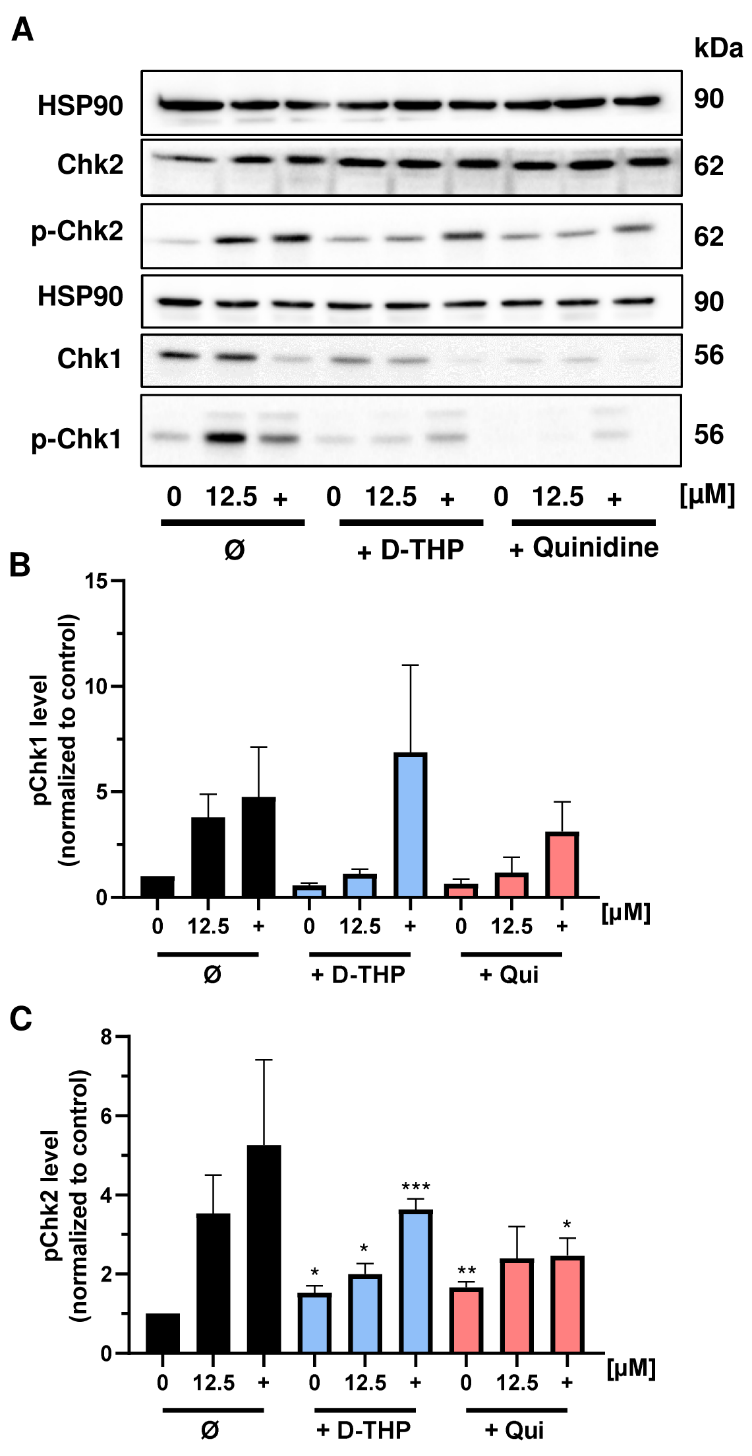
**

**SI Fig. 8:** **OCT1-inhibition and riddellinne-triggered DNA damage response (DDR). A** Representative western blots of (phosphorylated) CHK1 and CHK2 as downstream targets of the apical DDR kinases ATR and ATM after 24 h treatment with riddellinne. The genotoxic anticancer drug irinotecan was used as a positive control (+) and solvent as a negative control (0). Hsp90 served as additional loading control. **B** and **C** Densitometric evaluations of p-Chk1 (S345) and pCHK2 (Thr68) after 24 h incubation with riddellinne in HepG2-CYP3A4 cells. Unphosphorylated CHK1 and CHK2 served as loading controls. pCHK1 and pCHK2 levels relative to the loading control and normalized versus the negative control. Mean + SEM for three independent experiments (n=3). Statistical analyses were performed using unpaired two-tailed Students t-test with respect to the negative control. *P<0.05, **P<0.01, ***P<0.001, ****P<0.0001.
